# TIM3 in COVID-19; A potential hallmark?

**DOI:** 10.1016/j.heliyon.2024.e40386

**Published:** 2024-11-13

**Authors:** Mohammad Reza Zamani, Pavel Šácha

**Affiliations:** a Department of Cell Biology, Faculty of Science, Charles University, Prague, Czech Republic; b Institute of Organic Chemistry and Biochemistry, Czech Academy of Sciences, Prague, Czech Republic

**Keywords:** COVID-19, TIM3, Immune checkpoint, Biomarker, Virus, Infection

## Abstract

Coronavirus disease 2019 (COVID-19) is a highly contagious viral disease, caused by the severe acute respiratory syndrome coronavirus 2 (SARS-CoV-2). It can manifest as mild to severe flu-like and non-flu-like symptoms and signs, which are associated with immune dysfunction and increased mortality. The findings from COVID-19 patients imply a link between immune system abnormalities such as impaired T-cell responses or cytokine imbalances and increased risk for worse clinical outcomes, which has not been fully understood. Owing to the regulatory role of inhibitory immune checkpoints during COVID-19 infection, this review summarizes the available studies concerning the TIM3 as a relatively less characterized immune checkpoint in COVID-19 patients.

## 1 Introduction

Severe acute respiratory syndrome coronavirus 2 (SARS-CoV-2) first emerged in late 2019 in Wuhan, China. The spread of the virus resulted in a pandemic of coronavirus disease 2019 (COVID-19), which has caused millions of deaths worldwide. Although COVID-19 is no longer defined as a Public Health Emergency of International Concern, it remains a global threat, particularly due to the ongoing evolution of new SARS-CoV-2 variants [1].

Most people infected with SARS-CoV-2 experience mild or no symptoms, such as sore throat, dry cough, fever, and fatigue. However, some develop severe illness, including acute respiratory distress syndrome (ARDS), severe pneumonia, organ failure, or septic shock. Severe COVID-19 cases often involve immunological complications, such as lymphopenia, reduced T cell counts, increased proinflammatory cytokines, and impaired cellular immunity, partly due to T cell exhaustion and the overexpression of inhibitory checkpoint receptors (IC) [[2], [3], [4]].

An immune checkpoint (IC) is a group of inhibitory mechanisms in the immune system that control immune responses and preserve self-tolerance, ensuring that the immune system doesn't target the body's own tissues. These checkpoints involve interactions between receptors and ligands on the surface of immune cells, especially T cells, and can either amplify or dampen immune activity [5]. Many viruses and other pathogens can induce overexpression of IC receptors on immune cells, resulting in immune suppression and viral evasion [6]. Major ICs include cytotoxic T lymphocyte antigen-4 (CTLA-4), programmed cell death-ligand receptor 1 or 2 (PD-L1 or PD-L2), programmed cell death-1 (PD-1), lymphocyte-activation gene 3 (LAG-3), T cell immunoglobulin mucin 3 (TIM3), V-domain immunoglobulin suppressor of T cell activation (VISTA), T cell immunoreceptor with Ig and ITIM domains (TIGIT) and killer immunoglobulin-like receptors (KIRs) [7].

**Fig. 1:**
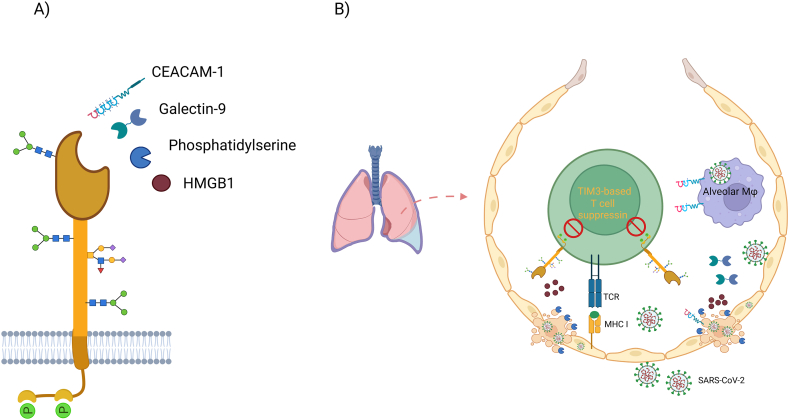
A) Schematic representation of TIM3 structure and its known ligands; Carcinoembryonic antigen-related cell adhesion molecule 1 (CEACAM1), Galectin-9 (Gal-9), Phosphatidylserine (PtdSer), High mobility group box 1 (HMGB1). B) A potential scenario of immune suppression in SARS-CoV-2-infected lungs. (Created in biorender.com).

TIM3 (Fig. 1A) is a relatively recently identified IC that has not been as extensively studied as other ICs like CTLA-4 and PD-1. Its dual roles—both inhibitory and stimulatory—along with its involvement in various diseases, have made TIM3 a focal point of research. It is particularly noted for its potential as a major mediator of T cell exhaustion in certain autoimmune disorders and in a range of preclinical cancer models [8].

During COVID-19 infection, studies have demonstrated that TIM3 is frequently upregulated, especially in severe cases. This upregulation is thought to impair the immune system's ability to eliminate the virus and regulate inflammation by promoting T cell exhaustion and weakening immune responses, potentially leading to worse disease outcomes. Additionally, it suggests that TIM3 could be explored as a measurable indicator of a biological state or condition (such as a biomarker) in COVID-19, and other viral infections [9], which warrants further investigation in future studies.

This review provides an overview of the latest research on TIM3 in COVID-19.

### 1.1 TIM3 as an inhibitory immune checkpoint

The immune system is vital for maintaining homeostasis and ensuring proper regulation of immunity. Self-tolerance is essential for preventing excessive or uncontrolled immune responses, which can otherwise result in inflammation, tissue damage, and autoimmune diseases. Various regulatory mechanisms, including inhibitory pathways, are in place to modulate the scale and intensity of immune reactions. Immune checkpoint (IC) receptors, present on a diverse array of immune and non-immune cells, play a key role in maintaining self-tolerance and modulating immune responses [10]. Several studies have identified correlations between elevated expression of IC receptors, including TIM3, and the severity of symptoms in COVID-19 patients [11].

TIM3, also known as hepatitis A virus cellular receptor 2 (HAVCR2), belongs to the TIM family, which was discovered in 2002 [12]. TIM3 is a type I transmembrane protein in the immunoglobulin (Ig) superfamily. It has an extracellular N-terminal immunoglobulin V (IgV) domain and a mucin-like domain abundant in glycosylation sites. Unlike other immune checkpoint proteins like PD-1, TIM3's intracellular C-terminal domain lacks canonical inhibitory signaling motifs. However, TIM3 signaling critically depends on the phosphorylation of two specific intracellular tyrosine residues, Tyr256 and Tyr263. This post-translational modification is crucial for TIM3's function and signaling pathways [13,14].

TIM3 was initially discovered on the surface of T helper (Th) 1 cells, but its expression has since been observed on a variety of other immune cells, including Th17 cells, CD8^+^ T cells, regulatory T (Treg) cells, natural killer (NK) cells, monocytes, macrophages, and dendritic cells (DCs). TIM3 interacts with several ligands, including galectin-9, phosphatidylserine, CEACAM1, and high mobility group protein B1 (HMGB1). The binding of TIM3 to its principal ligand, galectin-9, plays a key role in modulating T and NK cell responses by promoting cell death and inducing peripheral tolerance [13]. TIM3 may be co-expressed with other significant immune checkpoints, such as PD-1, on T cells. This co-expression can enhance the inhibitory effects on T cell activity and contribute to immune dysfunction in various conditions, including chronic infections and cancer [15].

In humans, the two primary sheddases responsible for cleaving the TIM3 ectodomain to release a soluble form are metalloproteinase 10 (ADAM10) and ADAM17 [16]. Lipopolysaccharide stimulation of CD14^+^ monocytes and T cell responses during graft-versus-host disease increases the shedding of TIM3 [17]. Additionally, individuals with untreated HIV exhibit increased shedding of TIM3 from CD8^+^ T cells. This shedding is associated with heightened immune dysfunction and may contribute to the overall immune suppression observed in HIV infection [18].

Mutations in *HAVCR2* are associated with hyperactivated T and myeloid cells, suggesting the role of TIM3 as an IC receptor [19,20]. Additionally, studies have revealed associations between some TIM3 single nucleotide polymorphisms (SNPs) and increased susceptibility to cancer and autoimmune diseases [21,22].

In cancer, TIM3 expression is a hallmark of the most exhausted subset of CD8^+^ T cells, and co-blockade of TIM3 and PD-1 can reactivate suppressed T cells, providing a significant anti-tumor effect in preclinical models [23].

### 1.2 TIM3 in viral infections

The possibility of excessive damage to the host cells during a course of a viral infection makes the controlled activation of the immunocytes very important. Several lines of evidence suggest that TIM3 levels are elevated and may play a role in viral infections.

In individuals with HIV, CD8^+^ T cell exhaustion is associated with increased expression of inhibitory receptors, such as TIM3 [24]. Additionally, effector CD4^+^ T cells also express elevated levels of TIM3, contributing to their dysfunction and to disease progression. TIM3 expression correlates positively with viral load and negatively correlated with active antiretroviral therapy. Co-blockade of PD-1 and TIM3 enhances the proliferation and cytokine production of HIV–specific CD8^+^ T cells [25].

In hepatitis B virus (HBV) infection, peripheral HBV–specific CD8^+^ T cells exhibit high expression of TIM3 along with other ICs, which is associated with their functional exhaustion. Additionally, CD4^+^ T cells in people with HBV infection have significantly elevated TIM3 compared to those of healthy individuals [26]. Blockade of ICs including TIM3 improves Th1 cytokine production in HBV infection [27]. Similarly, in chronic hepatitis C infection, development of hepatocellular carcinoma is linked to dysfunction of CD8^+^ and CD4^+^ T cells, which correlates with the elevated expression of several ICs, including TIM3.

During a primary influenza infection, expression levels of ICs including TIM3 was significantly increased on lung CD8^+^ T cells. The interaction of TIM3/Gal-9 can impair immune responses to influenza virus [28].

A recent study identified a TIM3-mediated immune evasion of influenza A virus subtype H1N1 through suppressing the RIG-I-type I interferon pathway. In macrophages, TIM3 signaling decreases RIG-I transcription and increases its proteasomal degradation. TIM3 blockade leads to upregulation of type I interferon, while silencing of RIG-I reverses this effect [29].

Co-infection of HIV patients with SARS-CoV-2 could potentially increase the risk of mortality or morbidity. A recent case report by Shahbaz et al. compared an HIV-untreated/SARS-CoV-2 co-infected patient with a SARS-CoV-2 mono-infected patient, both of whom were hospitalized on the same day and previously vaccinated for SARS-CoV-2. The study found higher expression of TIM3 in CD8^+^ cytotoxic T cells (CTLs), and soluble TIM3 in the plasma of the co-infection patient, as compared to mono-infection patient. Additionally, TIM3^+^ and PD-1^+^ CTLs from the co-infected patient exhibited an enhanced capacity for cytokine secretion *in vitro* compared to their negative counterparts, a phenomenon not observed in the mono-infected case [30].

## 2 TIM3 in COVID-19

### 2.1 TIM3 in PBMCs

The human peripheral blood mononuclear cells (PBMCs) include T and B cells (∼80 %), natural killer cells (∼10 %), monocytes and dendritic cells (∼10 %) [31]. PBMCs can be studied for various research applications including diagnostic, transplantation and cell therapy, drug screening and toxic reactions as well as identifying biomarkers and immunogenetic approaches. Although PBMCs cannot fully represent all aspects of the immune system, detailed immunophenotyping of these cells in company with other tissue-resident immune cells and analysis of the related soluble factors can generate certain valuable information. For instance, it has been shown that PBMC biomarkers can predict the response to immune checkpoint inhibitor therapy in metastatic breast cancer and the therapeutic efficacy of immunotherapy in non-small cell lung cancer [32].

Following the emergence of COVID-19 pandemic, some studies assessed the TIM3 expression in patient's PBMCs which are briefly mentioned here (Table 1).

**Table 1:** Summary of research on TIM3 in COVID-19 patients' samples.

Sample	Increased expression	Groups^a^	Ref.
**PBMCs**	1. CD8^+^ and CD4^+^ T cells	1. Severe	1 [33].
	2. NK, CD4^+^ and CD8^+^ T cells	2. Patients > Healthy	2 [34].
	3. CD4^+^ T cells	3. Critical and severe > Healthy	3 [35].
	4. CD8^+^ T cells	4. Critical > Non-critical & Healthy	4 [36].
	5. CD3^+^, CD4^+^ and CD8^+^ T cells	5. Case report	5 [37].
	6. NKT cells	6. Advanced > Recovered > Healthy	6 [38].
	7. CD4^+^ T cells	7. Severe > Mild & Asymptomatic	7 [39].
	8. CD4^+^ and CD8^+^ T cells	8. Extremely severe	8 [40].
	9. None	9. None	9 [41].
	10. CD4^+^ and CD8^+^ T cells	10. Post-acute > Asymptomatic	10 [42].
	11. IFN-γ^+^ SARS-CoV-2–specific CD4^+^ T	11. Recovered	11 [43].
	12. CD8^+^ T cells	12. Co-infected > Mono-infection	12 [36].
	13. CD8^+^ T subsets and total and central memory CD4^+^ T subsets	13. Infected mothers	13 [44].
**Tissue**	1. Lung autopsy	1. COVID-19 > non-COVID-19	1 [45].
	2. Skin biopsy	2. COVID-19 > non- COVID-19	2 [46].
**Serum/Plasma**	1. Post-acute COVID syndrome serum	1. Post-acute > Asymptomatic	1 [42].
	2. Severe patients' plasma	2. Severe > Non-severe & Healthy	2 [47].
	3. Hospitalized and discharged patients' serum	3. Hospitalized	3 [48].
	4. IMV-required patients' serum	4. IMV > Healthy	4 [49].
	5. Severe and ICU-admitted patients' serum	5. Severe > Mild	5 [50].
	6. Severe and mild patients' Serum	6. Severe > Mild	6 [51].
	7. ICU-admitted patients' plasma	7. ICU > Non- ICU	7 [52].
	8. Post-vaccine inflammatory syndrome	8. Case report	8 [53].
	9. Untreated-HIV and SARS-CoV-2 co-infection case plasma	9. Co-infection > Mono-infection	9 [30].

a An increasing trend in study groups is indicated by the '>' symbol.

One of the early and comprehensive studies comparing routine laboratory tests in patients with by Wang et al. on 65 patients with varying severity. Regarding TIM3, they found that extremely severe patients show an increasing trend in the expression of TIM3 on CD4^+^ and CD8^+^ T cells, however no significant difference was found among the groups [40]. Their study generally suggested an association between the CD4^+^ and CD8^+^ T cells hyperfunction and the pathogenesis of extremely severe patients.

Herrmann et al. measured very high expression of TIM3 on CD8^+^ and CD4^+^ T cells obtained from 20 people with COVID-19, compared to 13 healthy individuals. They observed a higher ratio of single-positive PD-1^-^ TIM3^+^ on CD8^+^ and CD4^+^ T cells over double-positive PD-1^+^ TIM3^+^ T cells. Moreover, TIM3 expression had a strong positive correlation with the activation status of the T cells (CD69, HLA-DR, and CD38) and the severity of the disease. TIM3 expression decreased during recovery from the infection [54].

Varchetta et al. studied NK, CD4^+^ and CD8^+^ T cells and found elevated TIM3 expression on these cells in 32 people with severe COVID-19 compared to 25 healthy individuals. Upon reanalyzing seven patients with acute COVID-19 who were discharged after 30–45 days, they observed a significant reduction in TIM3 expression on NK and CD8^+^ T cells [34]. Overexpression of TIM3 and CD69 on NK and T cells may point to the overactivation and exhaustion profile on both innate and adaptive immunity during sever infection.

Modabber et al. analyzed blood samples from 44 people with COVID-19, divided into moderate/severe and critical cases, and 16 healthy individuals. They found that the frequency of CD4^+^TIM3^+^ lymphocytes was significantly higher in both critical and severe patients when compared with healthy people, whereas there were no significant differences in the frequencies of CD4^+^ PD‐1^+^ and CD4^+^ CD39^+^ lymphocytes [35]. They also reported higher frequency of CD8^+^ TIM3^+^ lymphocytes in critical patients compared with noncritical and healthy controls.

A study examining TIM3 expression in 44 COVID-19 patients (17 critical and 27 non-critical) and 14 healthy individuals revealed that critical patients had significantly higher levels of CD8^+^ TIM3^+^ lymphocytes than non-critical patients and healthy individuals. Additionally, non-critical patients exhibited a significantly higher percentage of CD8^+^ TIM3^+^ cells compared to healthy individuals. Interestingly, there were no significant differences in CD8^+^ PD-1^+^ cell counts across all groups. The researchers also found that the numbers of CD8^+^ TIM3^-^ PD-1^-^ CD39^-^ cells were similar in both critical and non-critical patients but significantly lower than in healthy individuals [36]. These findings suggest that TIM3 and CD39 serve as exhaustion markers in CD8^+^ lymphocytes of SARS-CoV-2 patients.

A case study of a Caucasian married couple illustrated variations in TIM3 levels in COVID-19 patients. Patient 1 was a 60-year-old overweight male with type 2 diabetes, hypertension, and hemochromatosis type 1. Patient 2 was a 60-year-old woman without comorbidities. Both patients were reportedly infected simultaneously with the same dose of SARS-CoV-2 and initially experienced moderate and identical symptoms. However, patient 2 was later hospitalized due to worsening dyspnea. In contrast, patient 1 exhibited significantly higher expression levels of TIM3 on CD3^+^ cells, CD4^+^ and CD8^+^ T cells, as well as higher plasma Gal-9 levels, compared to patient 2 [37]. Although the cohort size is too small to draw definitive conclusions, the findings are consistent with other research and underscore the potential role of the TIM3 axis in the context of COVID-19.

Natural killer T (NKT) cells as a distinct subset of unconventional T cells were examined by Yang et al., using single-cell datasets from 1,462,702 cells across 81 advanced COVID-19 patients, 140 recovered patients, and 28 healthy individuals. They found a reduction in the total number of NKT cells in COVID-19 patients, but a significantly higher ratio of TIM3^+^ to TIM3^-^ NKT cells compared to healthy individuals, which was associated with disease progression. Additionally, convalescent patients had elevated levels of TIM3^+^ NKT cells compared to healthy individuals. The upregulation of TIM3 in NKT cells was linked to signs of exhaustion, increased apoptosis, and elevated expression of potential SARS-CoV-2 spike protein binding receptors, CD147 and CD26 [38]. These findings were in agreement with their previous studies showing the association between the overexpression of TIM3 and NKT cell apoptosis, activation and cytokines production in mice polymicrobial sepsis model [55].

Considering that individuals with no COVID-19 history and those with COVID-19 both have SARS-CoV-2–specific memory T cells that cross-react with common cold coronaviruses, Coulon et al. investigated the phenotype of cross-reactive SARS-CoV-2–specific CD4^+^ and CD8^+^ T cells targeting conserved epitopes shared with other common cold coronaviruses. They analyzed a cohort of 147 non-vaccinated COVID-19 patients with varying disease severities. Their study found that levels of exhausted SARS-CoV-2–specific CD4^+^ T cells co-expressing PD-1, TIM3, TIGIT, and CTLA4, as well as CD8^+^ T cells co-expressing these markers, were significantly higher in patients with fatal outcomes and those with severe symptoms who required intensive care, compared to individuals with mild or asymptomatic disease [39].

Post-acute COVID syndrome, also known as Long COVID or post-acute sequelae of COVID-19, is characterized by the persistence of physical and neuropsychiatric symptoms for more than 12 weeks. In a retrospective analysis of immune profiles from 21 COVID-19 patients, Bobcakova et al. found significantly elevated levels of PD-1, but not TIM3, on CD4^+^ and CD8^+^ T cells in survivors compared to those who died from COVID-19. Their data also revealed significant decreases in TIM3 expression, but not PD-1, on CD4^+^ and CD8^+^ T cells during the recovery phase in severe and critically ill patients [41]. Phetsouphanh et al. studied a cohort consisting of 31 individuals with post-acute COVID syndrome (symptoms lasting longer than 12 weeks), 31 asymptomatic individuals with SARS-CoV-2 infection, 25 individuals infected with common cold coronaviruses, and 13 healthy unexposed individuals. They found that TIM3 expression remained elevated on CD4^+^ and CD8^+^ T cells in patients with post-acute COVID syndrome at 3 and 8 months after infection, compared to asymptomatic individuals. However, this elevated TIM3 expression was only statistically significant for CD8^+^ T cells at 3 months. Interestingly, a subset of naive T and B cells expressing low levels of TIM3 was present in asymptomatic and healthy unexposed groups at 3 months but was absent in patients with post-acute COVID syndrome [42]. They also analyzed soluble TIM3 (sTIM3), which is discussed separately.

In the initial ex vivo activation study of SARS-CoV-2–specific T cells conducted by Hou et al., they demonstrated the maintenance of both humoral and cellular immune memory one year after infection in recovered COVID-19 patients. To assess immune memory to SARS-CoV-2, they recruited 26 patients with moderate COVID-19, 43 with severe COVID-19, and 9 with critical COVID-19, all one-year post-recovery. Following ex vivo stimulation of CD4^+^ T cells with a SARS-CoV-2–specific peptide pool and subsequent flow cytometry analysis, they observed that IFN-γ^+^ SARS-CoV-2–specific CD4^+^ T cells exhibited significantly higher levels of the activation marker HLA-DR and inhibitory receptors, including TIM3, compared to IFN-γ^-^ CD4^+^ T cells [43]. It is hypothesized that the loss of multifunctionality, along with increased expression of inhibitory receptors such as TIM3, in ex vivo activated SARS-CoV-2–specific CD4^+^ T cells may indicate a decline in immune memory over time.

Given the importance of maternal health and the increased risk of severe COVID-19 during pregnancy, a study by Vazquez-Alejo et al. compared the immune profiles of pregnant women with SARS-CoV-2 infection and their newborns to those of uninfected pregnant women and their unexposed newborns, both at birth and at 6 months postpartum. They found elevated levels of TIM3 and TIGIT in total, central memory, and effector memory subsets of both CD4^+^ and CD8^+^ T cells in infected mothers at both time points. Specifically, the co-expression of TIGIT and TIM3 (TIM3^+^ PD-1^-^ TIGIT^+^ LAG3^-^) was significantly increased in all CD8^+^ T cell subsets of SARS-CoV-2–infected mothers. For CD4^+^ T cells, the elevated co-expression of four exhaustion markers (TIM3^+^ PD-1^+^ TIGIT^+^ LAG3^+^) was significant in both total and central memory subsets. The study concludes that T cells in new mothers infected with SARS-CoV-2 during pregnancy show higher activation and exhaustion markers up to 6 months postpartum, whereas levels in their 6-month-old newborns remain normal [56].

One study, which is less consistent with most of the existing findings, reported a significantly elevated expression of PD-1 but not TIM3 on CD4^+^ or CD8^+^ cells between survivors and non-survivors. Bobcakova et al. retrospectively analyzed the immune profile of 21 cases divided into asymptomatic, mild/moderate, severe, critically ill, and deceased. They have shown data for significantly decreasing TIM3 expression during recovery of severe and critically ill patients on both CD4^+^ and CD8^+^ cells, but not for PD-1 [41]. It should be noted that for the expression of TIM3 and PD-1, they have only compared the survivors and non-survivors, which might be due to the small number of patients in each patient groups.

### 2.2 TIM3 in tissues

Most studies on COVID-19 immune responses have used low-throughput methods and focused primarily on peripheral blood samples, which provide limited information from a single tissue type. Collecting data from other affected tissues could enhance our understanding of the underlying immunological and pathophysiological mechanisms of COVID-19 (Table 1).

The first in situ study of the immune microenvironment in various tissues during COVID-19 was carried out by Wu et al. They performed bulk RNA sequencing on lung autopsy samples from 11 COVID-19 decedents and 3 non-COVID-19 decedents, utilizing Digital Spatial Profiling (DSP) and multiplex immunohistochemistry. Their findings revealed an association between TIM3 upregulation and severe clinical outcomes. Male lung tissues exhibited a more immunosuppressive signature and higher levels of inhibitory receptors, including TIM3, compared to female lung tissues. Additionally, there was a significant positive correlation between lung TIM3^+^ cells and age. The study also noted a higher presence of PD-1^+^ cells surrounding TIM3^+^ cells in men than in women, reflecting greater lymphocyte inhibition and suppression [45].

Cazzato et al. examined immunopathology in skin biopsies from eight COVID-19 patients with skin manifestations and eight individuals without COVID-19. While they found no statistically significant differences in the populations of CD4^+^ and CD8^+^ T cells between the groups, they observed higher levels of TIM3 and its potential ligand HMGB-1 in the skin samples from COVID-19 patients. This increased immunolabeling of TIM3 and HMGB-1 could contribute to the dysregulation of CD4^+^ and CD8^+^ T cells in the skin [46]. Given the elevated intracellular and extracellular levels of HMGB-1 and the presence of an immunosuppressive profile, especially in severe COVID-19 patients [57], it is hypothesized that the TIM3/HMGB-1 axis may contribute to dysfunctional immune responses and subsequent tissue manifestations, including skin abnormalities (Fig. 1B).

### 2.3 TIM3 in blood

The role of soluble molecules often lags behind that of their membrane-bound counterparts in research. In severe COVID-19 patients, T cell dysfunction is partly attributed to the imbalanced increase of both membrane-bound and soluble checkpoint molecules, which are crucial for regulating T cell responses and maintaining immune homeostasis. Therefore, further studies on soluble checkpoint molecules, such as sTIM3 (Table 1), could enhance our understanding of their role in immune regulation and the severity of COVID-19. Additionally, these molecules may serve as potential prognostic biomarkers or therapeutic targets for COVID-19.

In addition to evaluating cellular TIM3 expression, Phetsouphanh et al. assessed serum TIM3 levels 4 and 8 months after SARS-CoV-2 infection. Among 28 serum analytes, only six proinflammatory cytokines and sTIM3 were elevated in both the post-acute COVID syndrome and asymptomatic groups compared to the common cold coronavirus-infected and unexposed healthy groups. However, the difference was statistically significant only in the post-acute COVID syndrome group when compared to the common cold coronavirus-infected group. Additionally, sTIM3 levels were significantly lower at 8 months compared to 4 months in both the post-acute COVID syndrome and asymptomatic groups [42]. The findings reveal a continued abnormal and inflammatory immune profile even after mild-to-moderate COVID-19 infections. Furthermore, there is a positive correlation between elevated levels of both cellular and soluble TIM3.

Chen et al. measured plasma levels of sTIM3 in 55 participants, including 31 with non-severe COVID-19, 24 with severe COVID-19, and 31 healthy individuals. Their results show that elevated levels of sTIM3 in plasma are associated with disease severity, with significantly higher sTIM3 levels observed in patients with severe COVID-19 compared to healthy controls [47].

In two pooled prospective cohorts, Trøseid et al. [48] investigated circulating CD25 and TIM3 as markers of T cell activation and exhaustion, and their correlation with acute respiratory failure (RF), ICU admission, 60-day mortality, and pulmonary pathology. They found strong positive associations between elevated sCD25 and sTIM3 levels with both RF and ICU admission during hospitalization. While univariate analyses suggested both markers were linked to 60-day mortality, multivariate analyses showed that only sTIM3 remained significant. At the 3-month follow-up, increased sTIM3 levels were associated with pathological changes observed on chest CT scans. Notably, sTIM3 levels remained elevated throughout hospitalization and were still high at 3 and 12 months post-discharge, whereas sCD25 levels returned to levels comparable to healthy controls both during and after hospitalization.

Chavez-Galan et al. measured serum levels of sTIM3 in 105 COVID-19 patients, categorized into two groups based on their need for invasive mechanical ventilation (IMV) or supplemental oxygen via nasal cannula (NIMV), as well as 23 healthy controls. They observed that sTIM3 levels were elevated five-fold in COVID-19 patients compared to healthy controls. Additionally, significant differences in sTIM3 levels were noted between IMV and NIMV patients, suggesting that sTIM3 could serve as a potential biomarker to differentiate between these groups. The study also found positive correlations between sTIM3 levels, age, and duration of IMV. Notably, the data indicated that sTIM3 might offer greater sensitivity and specificity as a COVID-19 biomarker compared to sPD-L1 [49].

Kong et al. assessed serum levels of 14 soluble checkpoint receptors, including TIM3, in 109 hospitalized COVID-19 patients (44 with severe/critical disease, 60 with mild/moderate disease, and 5 asymptomatic). They found that 11 of these soluble checkpoint receptors, including sTIM3, were elevated in patients with severe or critical COVID-19 compared to those with mild or moderate disease. Among these, sTIM3 and three other soluble receptors were significantly associated with disease severity and the duration of ICU stay, indicating their potential as predictive biomarkers [50].

Avendaño‐Ortiz et al. analyzed over 20 soluble immunologic factors in the serum of 69 COVID-19 patients, including 29 with mild disease (outpatients and hospitalized without oxygen requirement), 26 with severe disease (hospitalized with oxygen requirement), and 14 who had died, as well as 15 healthy controls. Among these factors, three, including sTIM3, were found to positively correlate with disease severity. Elevated levels of these factors were observed in patients with mild COVID-19 compared to healthy controls and were significantly higher in severe and deceased patients compared to those with mild disease. sTIM3 levels were also correlated with age. In ex vivo experiments, blocking TIM3 with antibodies resulted in significantly increased proliferation and reduced apoptosis of CD4^+^ and CD8^+^ T cells derived from patients. The data further suggest that elevated levels of sTIM3 and sCD25 are strongly associated with mortality and the need for ICU care [51].

Ueland et al. investigated several plasma parameters, including sTIM3, as markers of T cell exhaustion in 39 adult COVID-19 patients (9 in the ICU and 30 outside the ICU). They found a significant association between elevated plasma levels of sTIM3 and the need for ICU admission. Additionally, sTIM3 levels were observed to significantly increase toward the end of the observation period among ICU patients [52].

Post-vaccine inflammatory syndrome, though rare, can occur following vaccination. A recent case report by Hsieh et al. detailed the immune profiles of two patients who developed systemic hyperinflammation with multiorgan involvement after receiving the ChAdOx1 nCoV-19 vaccine. Analysis of over 100 blood parameters revealed that, among the markers elevated in these patients compared to healthy controls, the following were notably increased: sTIM3, B-cell activating factor (BAFF), Resistin, urokinase plasminogen activator surface receptor (uPAR), platelet-derived growth factor (PDGF)-AB/BB, macrophage inflammatory protein-3-beta (MIP-3β), and interferon-inducible T-cell alpha chemoattractant (I-TAC) [53].

### 2.4 TIM3 ligands in COVID-19

Contrary to other “classic” checkpoint receptors such as PD-1, cytoplasmic tail of the TIM3 lacks inhibitory signaling motif. Although some *in vitro* studies indicate TIM3 as a co-stimulatory receptor in TCR signaling [58,59], however, TIM3 is widely accepted as a co-inhibitory receptor due to the link between loss-of-function mutations of *TIM3* gene and its polymorphisms with autoinflammatory conditions [60], as well as some promising clinical trials targeting TIM3 in cancer patients [61].

Four ligands have been identified that interact with the extracellular immunoglobulin V domain of TIM3 (Fig. 1A). Investigating these ligands in the context of COVID-19 may provide deeper insights into TIM3's role, its underlying immunological mechanisms, and its potential as a therapeutic target.

### 2.5 Galectin-9

Galectin-9 (Gal-9) was reported as the first natural ligand of TIM3. Gal-9 is a C-type lectin with two different carbohydrate receptor-binding domains which interact with carbohydrate moieties of membrane proteins. Many hematopoietic cells can express and secrete Gal-9. The interaction of TIM3/Gal-9 has shown to mediate the inhibition of immune responses in different cells [62]. On T cells, TIM3 undergoes oligomerization upon binding with Gal-9. This interaction leads to the release of BAT3 from the cytoplasmic tail of TIM3, which subsequently results in T cell inhibition and apoptosis [63].

Damaged lung epithelial cells secrete Gal-9 during viral infection including SARS-CoV-2 [64,65]. Bozorgmehr et al. found elevated levels of Gal-9 in the plasma of COVID-19 patients and proposed that Gal-9 acts as a Damage-Associated Molecular Pattern (DAMP), potentially exacerbating the cytokine storm by affecting neutrophils, monocytes, macrophages, and NK cells. Their model suggests that SARS-CoV-2–damaged lung epithelial cells release Gal-9, which then activates alveolar macrophages. This activation triggers a positive feedback loop, leading to increased production of proinflammatory cytokines and additional Gal-9 release from activated or apoptotic cells (Fig. 1B). Additionally, Du et al. demonstrated that Gal-9 facilitates SARS-CoV-2 binding and entry into human airway epithelial cells (AECs) in an angiotensin-converting enzyme 2 (ACE2)–dependent manner, enhancing the interaction between the viral spike protein and ACE2. Gal-9 treatment was also shown to increase SARS-CoV-2 replication in AECs cultured at an air-liquid interface (ALI) [66].

### 2.6 Phosphatidylserine

Phosphatidylserine (PS) molecules are normally present in the inner face of the cell membrane. They can however be exposed to the outer face, upon injury, infection or stimulation of the cell. PS is a surface marker for apoptotic cells and reported as the second natural ligand of TIM3. PS and TIM3 engagement on TIM3^+^ macrophages, dendritic cells and fibroblasts contributes to the engulfment of apoptotic material and antigen cross-presentation [[67], [68], [69]]. Conventional and tumor–infiltrating liver resident NK cells have shown to malfunction as a consequence of phosphorylation of TIM3 and impaired PI3K signaling upon binding to PS [70].

Althaus et al. found an upregulation of apoptotic markers in platelets from patients with severe COVID-19 who were admitted to the ICU. They observed that elevated phosphatidylserine (PS) externalization on platelets was linked to a higher likelihood of ICU admission. The study suggests that thromboembolic complications, which are associated with increased mortality in severe COVID-19, may be related to platelet apoptosis [71]. Moreover, Bohan et al. demonstrated through testing various human lung cell lines that the expression of TIM family receptors (TIM1 and TIM4) and TAM family receptors (AXL), which act as phosphatidylserine (PS) receptors, enhance SARS-CoV-2 infection. These receptors facilitate the virus's binding and entry into cells, as well as the internalization of virions, in an ACE2-dependent manner.

### 2.7 HMGB1

HMGB1 is an alarmin, which could be called the third identified natural ligand for TIM3. However, the exact binding site for TIM3/HMGB1 interaction is unknown. HMGB1 interacts with extracellular DNA released from dying cells, including those from tumors, thereby enhancing DNA recognition by Toll-like receptors (TLRs) [72]. It has been found that the interaction of TIM3 and HMGB1 on dendritic cells (DCs) can suppress the innate immune response to nucleic acids in the tumor environment [73]. Blocking TIM3 has shown to promote the activation of conventional DCs (cDC) in tumors [74], increase the responsiveness to cisplatin treatment (known to trigger the release of HMGB1) and the efficacy of DNA–based tumor vaccines in mouse models [73].

Infected or necrotic non-immune cells also release HMGB1 [75]. It is proposed that nuclear damage caused by catalytic effect of SARS-CoV-2 may promote the release of HMGB1 [75]. Furthermore, the active NF-κB signaling pathway due to the presence of high pro-inflammatory cytokines, can trigger monocytes and macrophages to release HMGB1 [76]. In COVID-19 patients, Chen et al. reported a positive correlation between high serum levels of HMGB1 and poor clinical outcomes and mortality. An *in vitro* study showed that HMGB inhibitors limit the upregulation of ACE2 and thus prevent SARS-CoV-2 infection [77]. Moreover, Chen et al., in 2004 had hypothesized a pathogenic role for HMGB1 in SARS-Cov1 [78].

### 2.8 CEACAM1

CEACAM1 glycoprotein (CD66a) is the most recent identified ligand for TIM3 [79]. It is co-expressed with TIM3 on the surface of monocytes, macrophages, DCs and activated T cells [80]. CEACAM1 can bind to TIM3 in both *cis* and *trans* configurations and mediate the inhibitory function of TIM3. CEACAM1/TIM3 axis has shown to play important role in autoimmunity, antitumor immunity and anti-viral responses [81]. Anti-TIM3 antibodies with *in vivo* antitumor efficacy block its interaction with CEACAM1 and PS [82].

CEACAM1 and CEACAM5 were reported to increase host susceptibility to bacterial infection following viral challenge in the human respiratory tract [83]. Huang et al. carried out the first systematic evaluation of CEA in COVID-19 patients using scRNA-seq data of bronchoalveolar lavage fluid (BALF) (accession no. GSE145926) [84] and PBMCs. The findings indicate that only CEACAM8-CEACAM6 but not CEACAM1 may contribute to the progression of COVID-19 by regulating the cell–cell communication in developing neutrophils and type II pneumocyte [85]. Sharif-Askari et al. conducted an in-depth analysis of immune receptor expression using in silico transcriptomic datasets from nasopharyngeal swabs, lung autopsies, bronchoalveolar lavage fluid (BALF), and blood samples from COVID-19 patients with varying degrees of severity. Their study revealed widespread expression of CEACAM1 in both myeloid and lymphoid immune cells across infections caused by Influenza A virus (IAV), respiratory syncytial virus (RSV), SARS-CoV-1, and SARS-CoV-2. Notably, CEACAM1 expression was found to be highest in cases of SARS-CoV-2 infection [86].

These findings may suggest PS receptor as a therapeutic target against SARS-CoV-2 [87].

## 3 Major mechanistic insights

As highlighted by various presented studies, the role of TIM3 in COVID-19 can be explored through two major mechanisms: TIM3-dependent T cell exhaustion and immune checkpoint synergy.

### 3.1 TIM3-dependent T cell exhaustion

In COVID-19, persistent viral presence leads to chronic antigen exposure, resulting in the upregulation of TIM3 on CD8^+^ T cells, which are essential for the immune response against viral infections. TIM3 binds to ligands such as Galectin-9 and CEACAM1 (Fig. 1), recruiting the phosphatase SHP2, which dephosphorylates key signaling molecules like Zap70 and LAT, thereby impairing T cell activation, cytokine production, and cytotoxicity—critical components of the antiviral defense [88]. Additionally, excessive phosphatidylserine on apoptotic-infected cells may contribute to T cell suppression by fostering a TIM3-based immunosuppressive environment (Fig. 1B). TIM3 signaling also inhibits the Akt-mTOR pathway, which is essential for T cell metabolism and survival [89].

### 3.2 Immune checkpoint synergy

Co-expression of TIM3 and PD-1, along with other immune checkpoints, on exhausted T cells during persistent viral infections, including SARS-CoV-2, results in a more profound inhibition of T cell responses due to the combined effects of their signaling pathways. This dual checkpoint engagement amplifies the suppression of T cell activation and antiviral functions, making it increasingly difficult for the immune system to control the virus. This synergy underscores the challenge of restoring T cell function in chronic infections like COVID-19 and highlights the need for therapeutic strategies that target multiple immune checkpoints simultaneously to overcome this profound immune suppression.

### 3.3 Other possible mechanisms

On NK cells, which are crucial antiviral components of the immune system, TIM3 expression is linked to decreased cytotoxic activity, impairing the clearance of infected cells, and potentially worsening the infection's severity. TIM3 ligands on NK cells, especially Galectin-9, contribute to this reduction in cytotoxicity. Moreover, TIM3 expression on both T cells and NK cells can paradoxically drive hyperinflammation by promoting cytokine release while also inducing immune exhaustion. This dual role of TIM3 in immune suppression and persistent inflammation can lead to tissue fibrosis, particularly in the lungs, a significant complication in severe COVID-19 cases [90]. The interplay between hyperinflammation and immune exhaustion, partly mediated by TIM3 ligands, contributes to the cytokine storm observed in severe COVID-19 cases.

## 4 TIM3 as a therapeutic target

Progressive exhaustion and functional dysfunction of the T cells represented by overexpression of inhibitory checkpoints appear to be a common condition during cancer and chronic infections. Currently, the major focus of the anti-TIM3 clinical trials is on the treatment of cancer. In this regard, there are only limited studies concerning the COVID-19 patients undergone PD-1/PD-L1 checkpoint therapy as the only relevant data at this time.

Anti-PD-1 antibody (nivolumab) administration to sepsis patients in a phase Ib randomized study reversed the depletion in CD4^+^ and CD8^+^ T cells without causing any symptom or sign of cytokine release syndrome [91]. A retrospective study failed to describe various cancer therapies including immunotherapy, as death risk factor by assessing 800 cancer patients who were COVID-19 infected in previous 40 days [92]. Two other similar studies on 5 and 7 patients under immunotherapy also failed to report any link between immunotherapy and COVID-19 mortality [93,94]. Herrmann et al. reported no severe disease course from two COVID-19 cohort patients who were previously treated with pembrolizumab (anti-PD-1 mAb) and rituximab (anti-CD20 mAb) [54]. However, Robilotti et al. reported a correlation between immune checkpoint therapy and more severe outcome in COVID-19 patients [95].

Up to now, no clinical trial is underway to treat COVID-19 through targeting TIM3. Considering some successful clinical trials targeting the TIM3 in cancer [96], theoretically it could be also possible to prevent the exhaustion and thus restore the effective T cell response to SARS-CoV-2 infection especially in patients with critical condition. On the other hand, such an intervention may lead to very severe inflammation with cytokine storm.

## 5 Conclusion and future directions

**Fig. 2:**
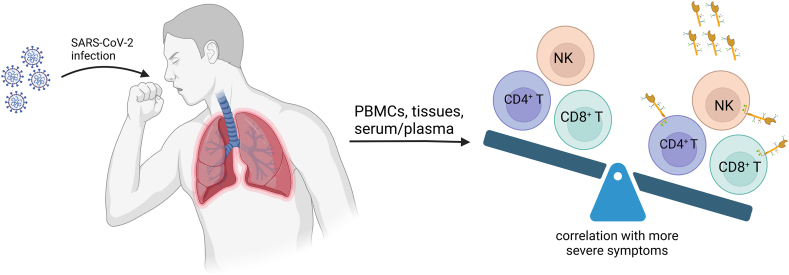
The upregulation of cellular and/or soluble TIM3 may contribute to the pathogenesis of COVID-19 by inducing immune system exhaustion. (Created in biorender.com).

Available studies suggest that cellular and soluble TIM3 could serve as a potential marker in COVID-19 patients, warranting further investigation concerning its actual mechanism and role (Table 1. & Fig. 2). Notably, TIM3 expression, but not PD-1, has been shown to correlate with the severity of the disease in some studies. Beyond disease severity, TIM3 expression and its plasma/serum levels have also been linked to factors such as age, gender, duration of invasive mechanical ventilation (IMV), ICU admission, and mortality. The observed significant reduction in TIM3 expression and soluble levels during recovery highlights the need for further research.

While no study has yet simultaneously evaluated TIM3 and all its ligands, separate investigations have shown upregulation of each of the four identified TIM3 ligands. This consistent upregulation suggests a potential immunological dysfunction and supports the hypothesis that TIM3 could be a relevant biomarker and/or therapeutic target.

Future research should focus on evaluating TIM3 expression in various immune cells, such as Treg cells, B cells, mast cells, monocytes, and their subsets. Each of these cell types plays a unique role in immune responses, and understanding how TIM3 is expressed across them could reveal its function in immune homeostasis and pathogenesis, particularly in COVID-19.

Additionally, evaluating TIM3 expression at various stages of COVID-19 and in different tissue samples from both autopsies and biopsies could provide a comprehensive understanding of its role. By comparing COVID-19 cases with other viral infections, researchers could determine whether TIM3 expression is specific to COVID-19 or part of a broader immune response to viral pathogens. This approach would also help clarify TIM3's role in disease progression, tissue-specific expression, and its potential as a therapeutic target.

Studying the impact of different COVID-19 vaccines on TIM3 expression and function, as well as examining germline mutations and gene polymorphisms in the TIM3 gene could provide further useful information about their potential correlations or causal effects o severity of COVID-19 and other viral infections.

Given the suppressive and inhibitory roles of TIM3, it would be valuable to investigate the effects of anti-TIM3 therapy in COVID-19 and other viral infections. While immune checkpoint inhibitors (ICIs) have shown promise in revitalizing exhausted T cells in COVID-19, the current studies exploring this approach are limited and inconclusive [95,97].

There are several issues including the notable limitations of the reviewed studies, which stem primarily from the urgent need for rapid approval and cohort setup during the pandemic. Most research is based on single-center studies with small sample sizes and data from a limited range of tissue types. Additionally, many studies are retrospective rather than prospective, and there is significant variability in methodologies and clinical focus. Despite these constraints, the findings consistently highlight TIM3 as a potentially valuable biomarker and therapeutic target in COVID-19 and potentially other viral infections.

While most research has focused on PD-1/PD-L1, which are well-established immune checkpoint targets in cancer therapy, TIM3 has emerged as an important factor. Notably, some studies have found that TIM3 expression or its soluble form correlates with COVID-19 severity, whereas PD-1 does not, highlighting the need for further investigation. Despite efforts to match age and gender among study participants, potential differences in TIM3 levels may also be influenced by other, as yet unknown, indirect effects. Additionally, although four ligands of TIM3 are known, the existence and role of other unidentified ligands cannot be ruled out.

Further studies are required to clarify the exact role of TIM3 and its ligands in COVID-19, assess its potential as a therapeutic target.

## CRediT authorship contribution statement

**Mohammad Reza Zamani:** Writing – review & editing, Writing – original draft, Software, Methodology, Funding acquisition, Conceptualization. **Pavel Šácha:** Writing – review & editing, Writing – original draft, Validation, Supervision, Software, Resources, Project administration, Methodology, Investigation, Funding acquisition, Conceptualization.

## Data availability statement

This article is a review and includes no research data.

## Declaration of generative AI and AI-assisted technologies in the writing process

During the preparation of this work the authors used chatgpt.com only to improve the language and readability. After using this tool, the authors reviewed and edited the content as needed and take full responsibility for the content of the publication.

## Funding

This study was supported by the project National Institute of Virology and Bacteriology (Programme EXCELES, ID Project No. LX22NPO5103) - Funded by the European Union - Next Generation EU, as well as the 10.13039/501100004240Academy of Sciences of the Czech Republic as part of the Strategy AV 21 Virology, Antiviral Therapy programme, 10.13039/501100001824Czech Science Foundation No. 24-10814S, and 10.13039/100007397Charles University Grant Agency project No. 390822.

## Declaration of competing interest

The authors declared that they do not have any commercial or associative interest that represents a conflict of interest in connection with the work submitted.
